# Integrating large language models in care, research, and education in multiple sclerosis management

**DOI:** 10.1177/13524585241277376

**Published:** 2024-09-23

**Authors:** Hernan Inojosa, Isabel Voigt, Judith Wenk, Dyke Ferber, Isabella Wiest, Dario Antweiler, Eva Weicken, Stephen Gilbert, Jakob Nikolas Kather, Katja Akgün, Tjalf Ziemssen

**Affiliations:** Center of Clinical Neuroscience, Department of Neurology, University Hospital Carl Gustav Carus Dresden, Technical University Dresden, Dresden, Germany; Center of Clinical Neuroscience, Department of Neurology, University Hospital Carl Gustav Carus Dresden, Technical University Dresden, Dresden, Germany; Center of Clinical Neuroscience, Department of Neurology, University Hospital Carl Gustav Carus Dresden, Technical University Dresden, Dresden, Germany; Else Kröner Fresenius Center for Digital Health, Technical University Dresden, Dresden, Germany; Else Kröner Fresenius Center for Digital Health, Technical University Dresden, Dresden, Germany; Fraunhofer Institute for Intelligent Analysis and Information Systems, Sankt Augustin, Germany; Else Kröner Fresenius Center for Digital Health, Technical University Dresden, Dresden, Germany; Fraunhofer Institute for Telecommunications, Heinrich Hertz Institute, HHI, Berlin, Germany; Else Kröner Fresenius Center for Digital Health, Technical University Dresden, Dresden, Germany; Else Kröner Fresenius Center for Digital Health, Technical University Dresden, Dresden, Germany; Center of Clinical Neuroscience, Department of Neurology, University Hospital Carl Gustav Carus Dresden, Technical University Dresden, Dresden, Germany; Center of Clinical Neuroscience, Department of Neurology, University Hospital Carl Gustav Carus Dresden, Technical University Dresden, Dresden, Germany

**Keywords:** Multiple sclerosis, large language models (LLMs), artificial intelligence, applications, disease management

## Abstract

Use of techniques derived from generative artificial intelligence (AI), specifically large language models (LLMs), offer a transformative potential on the management of multiple sclerosis (MS). Recent LLMs have exhibited remarkable skills in producing and understanding human-like texts. The integration of AI in imaging applications and the deployment of foundation models for the classification and prognosis of disease course, including disability progression and even therapy response, have received considerable attention. However, the use of LLMs within the context of MS remains relatively underexplored. LLMs have the potential to support several activities related to MS management. Clinical decision support systems could help selecting proper disease-modifying therapies; AI-based tools could leverage unstructured real-world data for research or virtual tutors may provide adaptive education materials for neurologists and people with MS in the foreseeable future. In this focused review, we explore practical applications of LLMs across the continuum of MS management as an initial scope for future analyses, reflecting on regulatory hurdles and the indispensable role of human supervision.

## Introduction

Digital tools and artificial intelligence (AI) in healthcare promise a new era in the management of multiple sclerosis (MS).^1^ MS presents unique challenges as a chronic and unpredictable disease, such as multidimensionality and heterogeneity of pathological findings and clinical manifestations, or an extremely variable disease course. Thereby, there is an urgent need for cutting-edge technologies to capitalize recent advancements in diagnostics, monitoring, and treatment for people with MS (pwMS).

In this evolving landscape, large language models (LLMs) stand as a key component of the field of generative AI with the capacity to enhance MS management with efficiency and already impressive precision.^2,3^ Since MS commonly affects younger individuals, notably women between 20 and 40 years, they may be especially receptive to an online second opinion provided through such platforms.^4,5^

LLMs have emerged demonstrating an unparalleled ability to generate texts that emulate human quality, adapting to required contexts and extending their utility to specialized fields in biomedicine.^2^ The applications of LLMs in managing MS are diverse and expanding, being possibly useful not only for MS specialists but for pwMS as well. We believe these applications could extend way beyond interactive chatbots, where pwMS can administrate their appointments or report symptoms. They could also include advanced educational tools and programs for text analysis or data extraction in setting of real-world analyses. These models are already explored for simulating and supporting clinical decision-making in other fields.^6^ In MS, integration of multimodal data obtained from various instruments and facilitated through AI algorithms with current medical knowledge may be used to support decision of immune or symptomatic treatments.^2^

An expanding literature highlights the utility of AI in MS, spanning from diagnosis to prognosis and monitoring, with a particular focus in imaging and multimodal data integration.^7^ However, there is still a substantial gap in the field of LLMs and their use in MS management remains relatively unexplored. Research is required for their use in direct patient care, MS-research, and education of stakeholders. In this focused review, we aim to approach these applications expanding the discourse on upcoming applications of LLMs within MS frameworks and with a clinical focus.^8,9^ Complementary to previous reports of LLMs in medicine, we examine the role of LLMs and how these could innovatively address complexities in the MS healthcare continuum.^8^ These complexities are not fully addressed by existing AI tools, considering the distinctive characteristics and varying disability progression of MS. We examine some of the unique challenges posed by MS, such as the heterogeneity of symptoms or the need for individualized treatment and how LLMs can approach these by offering innovative tools. Furthermore, we explore into the regulatory and ethical implications that accompany the implementation of these technologies, highlighting the importance of prioritizing patient safety. We aim to stimulate discussion about the potential of LLMs in MS, exploring their applications in leveraging AI in this complex disease.

## How do LLMs work?

LLMs employ complex algorithms that excel in producing remarkably human-like texts in the field of generative AI. Clusmann et al.^8^ provided a comprehensive analysis of the potential applications of LLMs within the medical field, accompanied by a useful glossary of computational terms. These models employ complex neural networks, often based on transformer architectures such as generative pre-trained transformer (GPT).^10^ This model, which relies on attention mechanisms, has revolutionized sequence transduction tasks by enhancing the ability to process contextual information without the need for recurrent or convolutional networks.

The capabilities of LLMs extend beyond simple question-answering as they can summarize, paraphrase, translate, or even transform verbal information.^11,12^ With the advent of several open-access LLMs, such as ChatGPT (OpenAI, San Francisco, California, USA), or Gemini (Google LLC, Mountain View, California, USA), both the general public and academic communities have started to recognize their potential for language-based applications.^13,14^ In the previous years, advances from GPT-3.5 to GPT-4o reflect significant improvements in performance, with enhanced understanding and improved responses from the latter. In addition, domain-specific models such as BioBERT, BioMegatron, and PubMedBERT have been developed, targeting the biomedical domain.^15–17^

LLMs use linguistic patterns and semantics through a self-attention mechanism that can recognize contextual nuances and the relationships between words and sentences.^8^ This understanding surpasses that of traditional machine learning models, which may rely on manual feature encoding or rule-based systems. By leveraging the self-attention mechanism, it enables more efficient and accurate predictions, making it an ideal foundation for developing advanced LLM applications in MS healthcare. Context here is also key: while the world “apple” can refer to a “fruit” or “technology” if accompanied by “edible” or “watch,” the word “attack” can refer to a “relapse” or “pseudo-relapse” depending on further data. A “lesion” may refer to an electrophysiological finding, optic nerve affection, or to histological characteristics. A “lesion” + “active” can have a different meaning if it involves “early” or “late” demyelinating or “mixed + inactive” as well (see Supplementary Figure). Based on this context, the probabilities in the transformer architecture are adjusted, influencing the produced outputs to ensure they are contextually accurate and relevant to the specific MS use-case or scenario.

**Figure 1.**
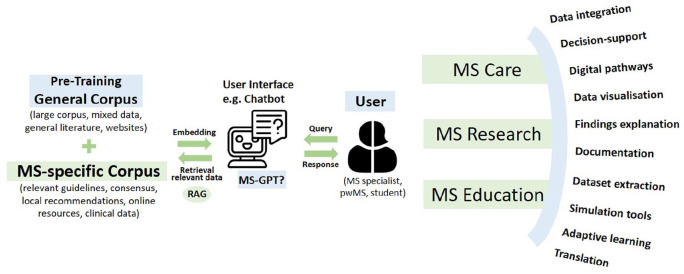
An integrated approach to large language models (LLMs) in multiple sclerosis (MS) management: a schematic representation. Use of an MS-specific corpus integrated to pre-training corpora using, for example, retrieval-augmented generation, where integration of multimodal data may enable several applications for MS specialists, people with MS (pwMS) or students in MS care, research, and education. Use-cases were intentionally not separated between fields as these intertwine in several cases. For example, data integration, simulation tools, or translation may be used for direct patient care, research, and education; documentation assistance can have a role primarily in care but also in education, dataset extraction and organization may be more relevant in research, although useful in care.

LLMs undergo pre-training on expansive datasets, or corpora, that include several interned-based sources to refine their language processing abilities. However, given that training corpora of currently available LLMs are often not disclosed and not fully known, it has been, to our knowledge, not yet been specifically tailored for MS-centered applications. Strategies for optimizing LLM output for specific tasks and domains are under discussion.^18^ For highly specific use-cases, LLMs can be fine-tuned on proprietary or local datasets. Recently, a group presented a fine-tuned model for detecting disease progression in MS with relatively little information from routine clinical practice.^19^ A particularly promising approach is the use of retrieval-augmented generation (RAG), which incorporates additional datasets during the response generation process.^18^ For instance, MS-specific literature—such as recent or regional guidelines, consensus papers, or scientific conference reports—can be used as source material in the form of targeted “chunks” of information to refine the foundational training of pre-existing models (see Figure 1). This enhancement can help to address issues such as the creation of erroneous content (“hallucinations”), reliance on outdated information, or responses lacking MS-specific knowledge by anchoring the LLMs’ responses in targeted, precise input as currently discussed exemplary in oncology.^12,20^

## LLMs in MS care

Processing and interpreting large datasets are fundamental to supporting clinical decisions in MS management. LLMs could integrate patient records, the latest research publications, and treatment protocols to provide evidence-informed guidance. Through neural networks and prediction models, they have shown proficiency in leveraging substantial medical knowledge and simulating complex reasoning skills typical of healthcare professionals.^21–23^ Presenting information in a user-friendly manner, such as through a chatbot interface, is particularly promising for MS specialists in routine care, where current evidence is updating practically on daily basis. Already with few data or inputs deep learning approaches have been able to predict disease courses.^19^ LLMs hold the potential to refine not just diagnosis and selection of disease-modifying therapies (DMTs), but also symptomatic treatments or rehabilitation. Moreover, LLMs can optimize information flow through digital pathways or monitoring platforms with cutting-edge MS-knowledge, enhancing communication channels with caregivers.^24^

For example, these models could serve as interactive interfaces to integrate data regarding risk for highly active disease course (e.g. description of relapses and their severity or MS lesion burden), hidden symptoms, demographics or inclusive financial aspects for treatment choice, summarizing not only data provided by MS specialists, but also directly from patients themselves. LLMs may also assist in creating personalized rehabilitation programs by analyzing patient preferences, conditions, or progress and suggesting modifications.

Effective communication is fundamental in MS care, where patient adherence can significantly influence treatment success and overall outcomes. A deep understanding of MS pathophysiology, symptoms, and treatment options is therefore essential. LLMs enable more digestible dialogues between MS specialists and pwMS. By translating difficult medical terminology into more accessible language, LLMs may assist pwMS in explaining their condition and therapies, as suggested by prior evaluations, for example, in a series of MS clinical scenarios.^25^ In addition, LLMs can tailor educational content to educational backgrounds and respond to common inquiries. For instance, brain or spinal magnetic resonance imaging (MRI) reports could be translated into layperson-friendly summaries. LLMs can also offer instant responses to patient questions when medical professionals are not immediately reachable. In an Italian cohort, a group of pwMS perceived responses provided by a chatbot to be higher empathetic than answers provided from specialists.^26^

The administrative load associated with documentation is well-known resource-consuming in medical domains, although few research is available in MS.^27^ However, LLMs may ease automating writing of clinical notes, communication with pwMS, and other necessary paperwork in ambulatory or stationary settings (e.g. writing tailored medical reports for family physicians, insurance entities, or social service entities), reducing the burden of tasks such as applying for assistive devices. This could begin with integrating speech-to-text technologies during consultation documentation. Further calculations of disability beyond the Expanded Disability Status Scale (EDSS), as already explored with machine learning, may be possible.^28^ Tests evaluating with MS-specific reports generated by LLMs are, however, not available. Moreover, LLMs can be used to streamline the process of obtaining insurance approvals for treatments by auto-generating necessary documentation and justifications based on data from medical records, for example, last EDSS, as data from symptoms documentation or symptomatic treatment may serve this purpose. Labels of approved DMTs could be checked with the patients records to assess potential drug interactions, contraindications, or appropriate dosing. This assistance may go beyond speeding up bureaucratic tasks, enabling MS specialists to dedicate more time for face-to-face patient interaction with potential to reduce medical expenses.

In efforts of implementing newer technologies into MS-healthcare, AI-based digital pathways are being integrated into a “digital MS twin” modeling the complexities of MS care within a virtual framework.^29^ These digital pathways could become integrated components to reflect and expand real-world clinical scenarios,^30^ improving involvement of pwMS, adherence, and outcomes.^31^

## LLMs in MS research

An expanding generation of knowledge in MS regarding the pathophysiology, treatment and prognostic of MS is occurring. Uses of LLMs in MS research share several aspects with the application in other medical fields or neurological diseases. LLMs can be useful by summarizing current findings, aiding MS researchers in accessing synthesized versions of scientific literature.^23,32^ Similarly, identification of emerging research opportunities, translational approaches, and innovative treatment strategies from other medical fields can be facilitated by LLMs. These can highlight the potential application of newer biomarkers used in other diseases, suggest novel therapeutic targets, and inspire cross-disciplinary approaches that could be beneficial in MS research. However, it is crucial to acknowledge that the usefulness of such content depends widely on the quality and sources of their training corpora, with the potential biases or conflicts of interest that may result from this. LLMs do not reflect their own output or weigh and balance arguments as humans, their output is based on calculations and predictions.

LLMs can particularly address complex MS-related datasets by streamlining the extraction and organization of unstructured textual data without the need for human reasoning, outperforming traditional machine learning methods.^19,33,34^ Although data from randomized controlled trials provide the highest levels of evidence, more research is required to optimize use of real-world input data from clinical routine settings.^35^ These, despite its raw and unformatted nature, provide insights beyond the setting of controlled trials.^36^ Given the variability in the real-world clinical presentation of MS, LLMs can assist researchers recognizing patterns and errors for improving data quality and consistency (e.g. inconsistent EDSS, lack of compliance in tests), integrating multimodal data sources or providing real-time feedback during data collection. Yet, the integration of “big data” presents challenges (e.g. heterogeneity and biases) that can be also present using AI.

Data on electronic health records, not only coming from discharge summaries, but also, from round notes or any written communications may serve as source of real-world data.^19,37^ A notable limitation in this area is the inherent subjectivity and potential bias from stakeholders in the primary documentation. Grading spasticity, describing eye movements, or characteristics of movement disorders is for neurologists or pwMS, still, relatively subjective. Nonetheless, beyond textual notes, data encompassed in different forms of language (including scales such as EDSS) and established clinical outcomes (including timed walk tests or assessments of visual acuity), or other types of data such as brain imaging, neurodestruction biomarkers, or digital sources (e.g. dynamometers, accelerometers, and smartwatches) could be incorporated.^38–40^

While certain models are already operational across different medical fields, a large unmet potential remains within MS research.^41,42^ For instance, research has shown the ability of a specifically tuned LLM to detect Alzheimer’s disease from medical records, surpassing the performance of human experts.^43^ Efficient identification of prodromal MS from unstructured real-world data (including apparently unrelated consultations or data from healthcare funding bodies) may also be possible to estimate real disease trajectories and investigate novel MS biomarkers.

In addition, LLMs could facilitate interactions among scientific collaborators. The adoption of LLM in drafting manuscripts or preparing grant proposals is a topic of active debate within various research communities.^44,45^ A position or statement from MS societies would likely be welcomed to address this aspect as a collective. We consider that questions regarding the appropriateness of LLM for draft generation, the boundaries of authorship and intellectual property, and whether LLM activities amount to those of a writing aid require careful consideration. AI systems hold the potential to streamline time-intensive research tasks, yet the generation of original content and conceptual thought may remain a human task.

## LLMs in MS education

Contrary to concerns of AI in education because of reduced learning or memorization, LLMs may offer innovative pedagogical approaches.^46^ Their integration into the medical curricula at an undergraduate level can provide instant access to high-quality medical knowledge, while including experiences of pwMS. Incorporation of latest MS corpora from medical guidelines, scientific literature, and medical conferences, for example, through RAG, would keep both students and healthcare professionals updated on best practices.^46^

Flexible LLMs could help creating interactive educational modules. Case-based learning, an established and efficacious educational strategy, may be further strengthened by LLMs, permitting stakeholders to virtually engage with the challenging diagnostic and therapeutic scenarios associated with MS. While existing online or digital models offer predetermined responses to learning scenarios, LLMs may provide a dynamic open interaction. LLM-based chatbots may reflect certain challenging nuances of symptom description by pwMS, as pain can range between somatic, visceral, and neuropathic types. This could enhance critical thinking and decision-making skills beyond multiple-choice or pre-established answers.

These models can be fine-tuned to “guide” users (e.g. students) in the desired direction.^18^ Cases may include managing various stages of MS, from initial diagnosis to handling advanced symptoms and complications. Thus, the open-ended depiction of disease course or the interpretation of diagnostic tests could be trained in an unsupervised free-response format. However, we should carefully consider individualized treatment decision-making and availability of resources, which may widely vary according to local resources or regulations and a ground truth or gold standard is frequently not available.^47^ Adapting the models to local characteristics and needs is important to avoid treatment gaps, as certain immune therapies may not be available or approved in specific regions. Moreover, treatment strategies may be considered in the customization, as the learning tool may adopt different approaches (e.g. hit hard and early vs escalation).

In addition, LLMs can change the development training exams and questions, as they could adjust to previous user results, although this application remains still untested in neurology and MS settings.^46^ Furthermore, LLMs can serve as instrumental resources preparing content, such as educational lectures or presentations. A request to “prepare a presentation discussing the diagnosis of secondary progressive MS” can yield a constructive template, which a junior lecturer can further refine.^27^

As mentioned above, patient education is fundamental in MS management. LLMs can play a significant role in disseminating understandable and accurate information to patients in a patient-friendly language. For instance, a newly diagnosed pwMS could interact with an LLM-based platform to understand their diagnosis or the suggested DMTs by the MS specialist. Management and interpretation of symptoms and decision support of possible relapse consultations may also serve in MS pathways or individual patient journeys.^48^ LLMs can be prompted and adjusted to deliver personalized educational content adapted to individual profiles, including age, background, or specific symptoms, while also considering cognitive capacities of pwMS. This may include interpreting clinical trial findings, negative outcomes from phase III studies, or information about complementary or alternative therapies. Digital health literacy is necessary also in MS, as pwMS could take advantage of digital resources for their health.

## Challenges and regulations

While LLMs hold immense potential in healthcare and MS management, their deployment in areas requiring medical interpretation must be approached with careful oversight and human involvement. If AI systems are used in complex clinical scenarios for applications in diagnosis, treatment, monitoring, or prognosis of MS, they are subject to stringent regulation to ensure they are utilized safely and effectively.^49,50^ The integration of LLMs into MS poses tangible challenges common to other medical fields, including risks of disseminating misinformation, privacy breaches, inherent biases from training datasets, and the danger of abuse.

Ensuring that the employment of patient data through LLMs adheres to data protection regulations is imperative, as the confidentiality and integrity of sensitive health information are non-negotiable. Leakage of such data remains a concerning threat that must be addressed.^50^ Federated learning, as an example, is an emerging collaborative approach that could enable the contribution between MS centers to a shared MS research model without exchanging sensitive patient data, maintaining privacy and data ownership.^51^

The European Union’s recent enactment of the first AI Act is a landmark development, categorizing AI systems according to their potential risk levels and delineating responsibilities for both developers and users.^52^ Unsurprisingly, AI systems with the potential to significantly impact healthcare—where the stakes can involve life-or-death outcomes and substantial financial implications—are identified as high risk and subjected to rigorous regulative measures. This legislation underscores the importance of transparency and risk management throughout the AI development lifecycle.

Addressing specific challenges in incorporating LLMs into MS management necessitates thoughtful strategies, as laid out in Table 1. Many of these considerations—reinforcing ethical development, bolstering cyber-secure infrastructures, and promoting data stewardship—are encapsulated within the framework of the European Union’s AI Act.

**Table 1.** Navigating Challenges and Pioneering Solutions for Enhancing AI and LLM in MS Management.

Challenges	Approaches
Multicenter data availability and with heterogeneous data characteristics and collection method	Establishment of data-sharing agreements and collaborations (e.g. federate learning). Open-data initiatives
Data bias and representation, bias propagation	Use of diverse datasets involving pwMS from diverse characteristics and backgrounds can enhance training, refinement, and relevance of LLMs
Lack of defined “ground truth” in dynamic MS advances and regional differences	Periodical updates of LLMs, incorporation of diverse international perspectives in training and testing of models. Real-time employment of data-retrieval strategies (e.g. RAG)
Limited contextual understanding and nuanced judgment	Training enhancement and customization, (e.g. fine-tuning)
Over-reliance in AI technologies (automation bias)	Human oversight and validation to complement AI-driven decisions
Hallucination	Robust anomaly detection mechanisms and model recalibration strategies (e.g. feedback loops, early warning, adaptive learning, or regular updates through an MS societal task force)
Data privacy	Robust data security measures in training and use of LLMs (e.g. encryption, anonymization, and access controls to protect sensitive data)
Lack of validation studies	Rigorous real-world validation studies across diverse settings
Unknown structure and training corpus in closed-source models	Advocate for transparency and regulatory scrutiny of proprietary algorithms (e.g. EU AI act)
Technical infrastructure with restricted access across shareholders from different backgrounds	Scalable cloud computing resources for widespread accessibility
Fragmentation and inefficiency in development due to parallel working groups	Utilization of multicenter joint platforms, task forces or working groups; workshops and webinars

pwMS: people with MS; MS: multiple sclerosis; LLM: large language model; RAG: retrieval-augmented generation; AI: artificial intelligence; EU: European Union.

The widespread adoption of LLMs in patient treatment will require balancing their flexibility with ethical, legal, and procedural safeguards. As such, safe integration of this technology into MS management will rely on an ongoing dialogue between developers, healthcare professionals, pwMS, regulators, and the wider community, with safety as the foundational priority.

## Vision: integrating large multi-modal models in MS care

Although research and use of LLMs in MS are still in early stages, other established AI technologies such as machine learning algorithms for imaging and pattern recognition are already more developed and tested, being possibly suitable for use in the coming future.^53^ As previously noted, these technologies may complement the capabilities of LLMs. Multi-modal models could interpret data from different forms, encompassing several aspects of the disease and even making predictions in real-world scenarios.

In neuroimaging, non-LLM-based AI algorithms are already showing considerable promise in enhancing the understanding and management of MS.^19,54^ These algorithms offer capabilities for identifying and monitoring MS lesions, as well as quantifying changes in brain tissue, such as regional or global atrophy, over time. Some of these AI-driven models are currently near to approval process, heralding their potential integration into clinical practice for MS management.^55,56^ Furthermore, AI applications in neuroimaging are expanding, with research exploring the identification of novel biomarkers, supporting differential diagnoses of white matter anomalies, and predicting disease progression through imaging data. In addition, optical coherence tomography (OCT) leverages AI to augment the detection of MS, reflecting the versatility of AI in different imaging modalities.^57^

AI’s proficiency identifying longitudinal dynamics in biomarkers presents an exciting opportunity for early detection of disease progression or the efficacy of DMTs. Beyond imaging, data from wearable technologies offer real-time insights into physical parameters, including movement patterns, heart rate variability, sleep quality, and activity levels, which are especially interesting given the symptoms such as fatigue and mobility in pwMS.^58^

Recognizing subtle changes, particularly in early stages of MS, is challenging. AI provides a refined tool to detect and interpret these nuances. In synthesizing these insights, the integration of multi-modal data through AI together with LLMs can significantly enhance patient management across all spectrums of MS.

Further research and efforts are needed to assess the potential applications of LLMs, as commented above. Combining diverse data streams with clinical information systems supported by LLMs stands to transform the landscape of MS care, research, and education. By integrating the strengths of various AI tools, a holistic approach is possible, one that delivers a comprehensive, patient-centric care of pwMS. This may offer a platform that not only involves answering questions on a chatbot, but also aims for a personalized disease management. As we move forward, increasing applications of generative AI seem to be possible in the near future for a novel digital MS management.

## Supplemental Material

sj-jpg-1-msj-10.1177_13524585241277376 – Supplemental material for Integrating large language models in care, research, and education in multiple sclerosis management Supplemental material, sj-jpg-1-msj-10.1177_13524585241277376 for Integrating large language models in care, research, and education in multiple sclerosis management by Hernan Inojosa, Isabel Voigt, Judith Wenk, Dyke Ferber, Isabella Wiest, Dario Antweiler, Eva Weicken, Stephen Gilbert, Jakob Nikolas Kather, Katja Akgün and Tjalf Ziemssen in Multiple Sclerosis Journal
