# Supplementary figures and images for: Integrating large language models in care, research, and education in multiple sclerosis management
Source: Mult Scler. 2024 Sep 23;30(11-12):1392–401. doi: 10.1177/13524585241277376 (PMC11514324; doi:10.1177/13524585241277376)

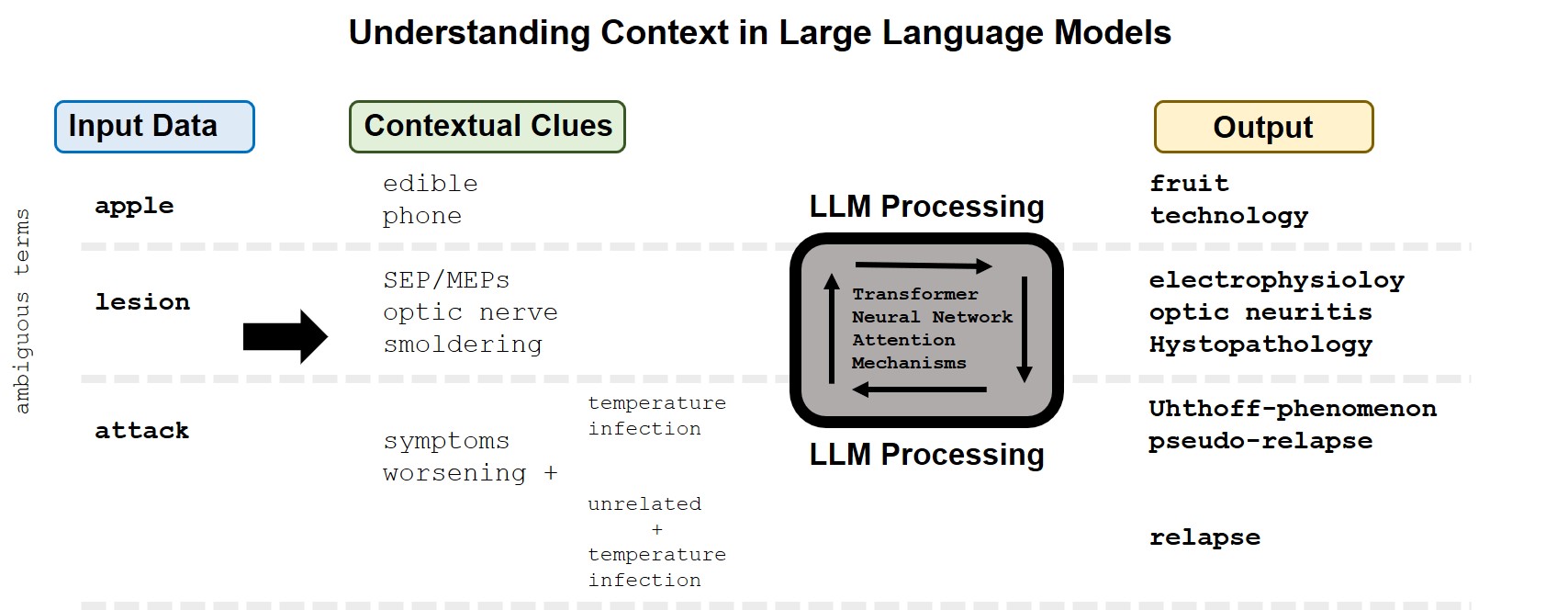

Supplement: sj-jpg-1-msj-10.1177_13524585241277376 – Supplemental material for Integrating large language models in care, research, and education in multiple sclerosis management [file sj-jpg-1-msj-10.1177_13524585241277376.jpg]
